# Knowledge and Attitude Toward Modes of Delivery and Possible Complications Among Women in Al-Baha Region, Saudi Arabia

**DOI:** 10.7759/cureus.59988

**Published:** 2024-05-09

**Authors:** Eman A Keshk, Ghadi S Alghamdi, Maali A Alghamdi, Manar S Alzahrani, Raghad M Alzahrani, Afaf S Alzahrani, Reema S Alzahrani

**Affiliations:** 1 Obstetrics and Gynecology, Faculty of Medicine, Suez Canal University, Ismailia, EGY; 2 Obstetrics and Gynecology, Faculty of Medicine, Al-Baha University, Al-Baha, SAU; 3 Medicine, Al-Baha University, Al-Baha, SAU

**Keywords:** cesarean section, vaginal delivery, modes of delivery, knowledge, attitude

## Abstract

Background: The experience of childbirth significantly influences women's perspectives and attitudes, which are shaped by whether their experiences were positive or negative. This study aims to assess knowledge and attitudes regarding childbirth methods and potential complications among women in Al-Baha, Saudi Arabia.

Methods: This cross-sectional study was conducted in the Al-Baha region of Saudi Arabia with 500 participants. Data collection was facilitated through online surveys. The survey was responded to by women residing in Al-Baha of reproductive age (18-45), including those who had given birth vaginally or via cesarean section within the past five years. The questionnaire covered sociodemographic aspects and assessed knowledge and attitudes toward vaginal delivery, cesarean section, and their respective complications.

Results: The study encompassed 500 participants, predominantly under 36 years of age (224 participants, 44.8%). A majority were married (355 participants, 71%) and held a university degree (358 participants, 71.6%). Notably, half of the participants were unemployed, and 365 (65.4%) were multiparous. Preferences for childbirth methods showed that 296 women (59.2%) favored vaginal delivery, while 100 women (20%) preferred cesarean section. Regarding knowledge about childbirth, 200 participants (40%) rated their knowledge as very good.

Conclusion: The study reveals a preference for vaginal delivery among the women surveyed, with over half possessing adequate knowledge about postpartum complications. Women with only a diploma or expressing a preference for cesarean section displayed lower knowledge levels about these complications. It is recommended that medical professionals provide comprehensive information about various childbirth methods and their complications, indications, and benefits to expectant women.

## Introduction

A cesarean section (C-section), the most frequent abdominal surgery, has various indications including fetal distress, advanced maternal age, labor arrest, and malpresentation [1,2]. The delivery experience significantly influences women's perspectives and attitudes, which vary based on whether the experience is positive or negative [3]. The World Health Organization (WHO) advises that C-section deliveries should not exceed 10-15% of the population. Despite this, the global prevalence of C-sections is rising and is projected to reach 29% by 2030 [4]. C-sections are associated with a higher risk of venous thrombosis, urinary tract injuries, hemorrhage, anesthesia-related complications [5], postpartum infections, pain, headaches, maternal death, and postpartum depression [6,7]. It has been shown that women who are informed about the requirements and risks of C-sections are less likely to choose elective births. Reducing unnecessary C-sections through education and awareness may decrease maternal and infant morbidity and mortality [8,9]. The increasing C-section rates have significant implications for healthcare systems, such as increased costs and resource utilization. C-sections require specialized facilities, skilled healthcare providers, and postoperative care, thus burdening limited healthcare resources [10,11]. Moreover, the extended recovery time and potential complications of C-sections can lead to longer hospital stays and increased healthcare expenses [12]. Therefore, it is crucial to explore strategies that promote evidence-based practices and ensure the judicious use of C-sections, aiming to optimize resource allocation and enhance the efficiency of obstetric care delivery. This study aimed to assess knowledge and attitudes regarding childbirth methods and potential complications among women in Al-Baha, Saudi Arabia.

## Materials and methods

Study design 

A cross-sectional study was conducted from June 2023 to August 2023 to assess the level of knowledge and attitude toward modes of delivery and possible complications among women in the Al-Baha region, Saudi Arabia. 

Study population and sampling

The study population for this research consisted of women residing in Al-Baha, aged 18-45 years, who had given birth either vaginally or via C-section within the past five years and met the inclusion criteria. Participants who were not willing to participate or fell outside the reproductive age range (below 18 or above 45) were excluded from the study.

The sample size was determined using the Raosoft website [13]. With a confidence level of 95% and a margin of error of 5%, the calculated sample size for this study was a minimum of 385 participants. A total of 500 participants were selected using a random sampling technique to account for potential non-responses or incomplete data. Additionally, participants were identified as visitors to primary healthcare centers (PHCs) and hospitals.

Data collection and instrument 

Data were collected through a predesigned, validated online questionnaire completed by women residing in the Al-Baha region. The questionnaire consisted of four sections (Appendices). Section 1 of the survey collected information on the participants' demographic characteristics and assessed their knowledge and attitudes regarding birth methods and potential complications. The demographic information included age group, marital status, education level, and occupation. Participants were also asked about their pregnancy history, preferred birth method, monthly family income, and self-evaluation of knowledge about birth methods and possible complications. Section 2 of the survey aimed to assess participants' knowledge and attitudes toward vaginal birth. It utilized a Likert scale to gauge participants' responses. Eight questions covered various aspects of vaginal delivery, such as its natural acceptability, impact on maternal happiness and emotional bonding, recovery period, avoidance of surgical risks, long-term benefits, preference to avoid abdominal scarring, and comparison of complications with C-section, on a scale ranging from "Strongly agree" (5 points) to "Strongly disagree" (1 point). A grading system was employed, assigning point values to each response option. The positive attitude score was calculated by summing the points for each question, with a threshold of 19 or more indicating a positive attitude toward vaginal delivery. Section 3 of the survey consisted of 10 questions on a Likert scale, aimed at assessing participants' knowledge regarding C-section and their attitudes toward it. The questions covered various aspects related to C-section, including its perceived superiority, preference to avoid stress and pain, perceived health benefits, additional procedures, prevention of complications, and decision-making factors, on a scale ranging from "Strongly agree" (5 points) to "Strongly disagree" (1 point). A grading system was employed, assigning point values to each response option. The positive attitude score was calculated by summing the points for each question, with a threshold of 24 or more indicating a positive attitude toward C-section. Conversely, a negative attitude was indicated by a score of less than 24. Section 4 of the survey was dedicated to assessing participants' knowledge of postpartum complications. The section consisted of seven questions on a Likert scale, ranging from "Strongly agree" (5 points) to "Strongly disagree" (1 point). The questions covered various aspects of postpartum complications, including infections such as endometriosis, deep vein thrombosis, bleeding, hematoma in the bladder after a C-section, wound infection after a C-section, complications resulting from medical errors such as uterine perforation, and complications such as acute cholecystitis. Participants' responses were graded using a point system, with higher points assigned to responses indicating greater agreement with the statement. The knowledge score was calculated by summing the points for each question. A score of 4 or higher indicated sufficient knowledge of postpartum complications, while a score of less than 4 indicated insufficient knowledge.

Statistical analysis

The collected data were analyzed using IBM SPSS Statistics for Windows, Version 29.0 (Released 2023; IBM Corp., Armonk, New York, United States). Descriptive statistics summarized the demographic characteristics of participants (age groups, educational level, city of residency, and occupation). The association between participants' characteristics and their knowledge of postpartum complications, as well as their attitudes toward vaginal delivery and C-section, were examined using the chi-squared test and the Fisher-Freeman-Halton exact test extension. P-values less than 0.05 were considered statistically significant.

Research ethics 

This study received approval from the Scientific Research and Ethics Committee of the Faculty of Medicine, Al-Baha University, Al-Baha, Saudi Arabia (approval number: REC/OB/BU-FM/2023/49, dated June 12, 2023). All participants voluntarily engaged in the survey and were informed of the study's purposes and objectives. Informed consent was obtained for anonymous participation, ensuring the confidentiality of responses.

## Results

The study involved a total of 500 participants. Among them, the majority, 224 participants (44.8%), were under 36 years old. A significant proportion, 355 (71%), were married women, and 358 (71.6%) of the women held university degrees. Additionally, the study revealed that half of the participants were unemployed and 365 (65.4%) were multiparous. Regarding delivery preferences, 296 women (59.2%) preferred vaginal delivery, while 100 (20%) opted for a C-section. In terms of knowledge, 200 participants (40%) rated their knowledge as very good (Table 1).

**Table 1:** Demographic characteristics of participants N: number; %: percentage

n=500		N	%
Age (in years)	18-25	140	28.0%
	26-30	41	8.2%
	31-35	43	8.6%
	36-40	71	14.2%
	41-45	85	17.0%
	>45	120	24.0%
Marital status	Single	123	24.6%
	Married	355	71.0%
	Divorced/widowed	22	4.4%
Education	Below high school	13	2.6%
	High school	78	15.6%
	Diploma degree	51	10.2%
	University degree	358	71.6%
Employment status	Unemployed	253	50.6%
	Employed	247	49.4%
Family income	<5000	43	8.6%
	5000-10000	173	34.6%
	>10000	284	56.8%
Parity	Nullipara	144	28.8%
	1	29	5.8%
	≥2	327	65.4%

Table 2 details the pregnancy data of the participants. Of the women, 296 (59.2%) preferred vaginal delivery, and 100 (20%) favored C-section. Concerning knowledge rating, 200 (40%) participants rated their knowledge as very good and 166 (33.2%) as good. A smaller proportion rated their knowledge as excellent and poor (18.2% and 6.8%, respectively).

**Table 2:** Pregnancy data of participants N: number; %: percentage

n=500		N	%
Preferred mode of delivery	Vaginal delivery	296	59.2%
	Cesarean section	100	20.0%
	Neutral	104	20.8%
Participants' perceived knowledge rating	Poor	43	8.6%
	Good	166	33.2%
	Very good	200	40.0%
	Excellent	91	18.2%

Table 3 illustrates the total knowledge scores and attitudes of the participants. Over half (55.6%) possessed sufficient knowledge about postpartum complications. Only five participants (1%) exhibited a negative attitude toward vaginal delivery, while 118 (23.6%) held a negative view of C-section.

**Table 3:** Total knowledge scores and attitudes of participants N: number; %: percentage

n=500		N	%
Knowledge about postpartum complications	Insufficient	222	44.4%
	Sufficient	278	55.6%
Attitude toward vaginal delivery	Negative attitude	5	1.0%
	Positive attitude	495	99.0%
Attitude toward cesarean section	Negative attitude	118	23.6%
	Positive attitude	382	76.4%

Table 4 illustrates the factors associated with the participants' knowledge scores. Notably, participants holding a university degree exhibited a significantly higher awareness of postpartum complications compared to those with only a diploma or lower education level (*p*=.022). Additionally, the data indicate that participants favoring C-section showed a substantially lower understanding of postpartum complications than those preferring vaginal delivery (*p*=.005).

**Table 4:** Factors associated with knowledge scores of participants *P-values based on the chi-squared test; statistical significance at *p*<0.05

n=500		Knowledge about postpartum complications				
		Insufficient		Sufficient		
		N	%	N	%	*p*-value
Age (in years)	18-25	59	26.6%	81	29.1%	0.192
	26-30	18	8.1%	23	8.3%	
	31-35	19	8.6%	24	8.6%	
	36-40	24	10.8%	47	16.9%	
	41-45	38	17.1%	47	16.9%	
	>45	64	28.8%	56	20.1%	
Marital status	Single	52	23.4%	71	25.5%	0.769
	Married	159	71.6%	196	70.5%	
	Divorced/widowed	11	5.0%	11	4.0%	
Education	Below high school	6	2.7%	7	2.5%	0.022^*^
	High school	32	14.4%	46	16.5%	
	Diploma degree	33	14.9%	18	6.5%	
	University degree	151	68.0%	207	74.5%	
Employment status	Unemployed	110	49.5%	143	51.4%	0.675
	Employed	112	50.5%	135	48.6%	
Family income	<5000	22	9.9%	21	7.6%	0.641
	5000-10000	75	33.8%	98	35.3%	
	>10000	125	56.3%	159	57.2%	
Parity	Nullipara	65	29.3%	79	28.4%	0.541
	1	10	4.5%	19	6.8%	
	≥2	147	66.2%	180	64.7%	
Preferred mode of delivery	Vaginal delivery	136	61.3%	160	57.6%	0.005^*^
	Cesarean section	31	14.0%	69	24.8%	
	Neutral	55	24.8%	49	17.6%	
Participants' perceived knowledge rating	Poor	23	10.4%	20	7.2%	0.115
	Good	74	33.3%	92	33.1%	
	Very good	94	42.3%	106	38.1%	
	Excellent	31	14.0%	60	21.6%	

Table 5 shows the factors influencing participants' attitudes toward vaginal delivery. It demonstrates that those favoring C-section were significantly more inclined to hold negative views about vaginal delivery (*p*=.007). This suggests a greater likelihood of adverse attitudes toward vaginal delivery among C-section proponents. Other factors such as age, marital status, educational level, employment status, family income, and parity did not show a significant relationship with attitudes toward vaginal delivery.

**Table 5:** Factors associated with attitudes of participants toward vaginal delivery *P-values based on the chi-squared test and the Fisher-Freeman-Halton exact test; statistical significance at *p*<0.05

n=500		Attitude toward vaginal delivery				
		Negative attitude		Positive attitude		
		N	%	N	%	*p*-value
Age (in years)	18-25	1	20.0%	139	28.1%	0.137
	26-30	2	40.0%	39	7.9%	
	31-35	0	0.0%	43	8.7%	
	36-40	1	20.0%	70	14.1%	
	41-45	1	20.0%	84	17.0%	
	>45	0	0.0%	120	24.2%	
Marital status	Single	0	0.0%	123	24.8%	0.469
	Married	5	100.0%	350	70.7%	
	Divorced/widowed	0	0.0%	22	4.4%	
Education	Below high school	0	0.0%	13	2.6%	0.081
	High school	3	60.0%	75	15.2%	
	Diploma degree	0	0.0%	51	10.3%	
	University degree	2	40.0%	356	71.9%	
Employment status	Unemployed	4	80.0%	249	50.3%	0.373
	Employed	1	20.0%	246	49.7%	
Family income	<5000	0	0.0%	43	8.7%	0.600
	5000-10000	3	60.0%	170	34.3%	
	>10000	2	40.0%	282	57.0%	
Parity	Nullipara	1	20.0%	143	28.9%	0.283
	1	1	20.0%	28	5.7%	
	≥2	3	60.0%	324	65.5%	
Preferred mode of delivery	Vaginal delivery	0	0.0%	296	59.8%	0.007^*^
	Cesarean section	3	60.0%	97	19.6%	
	Neutral	2	40.0%	102	20.6%	
Participants' perceived knowledge rating	Poor	0	0.0%	43	8.7%	0.493
	Good	2	40.0%	164	33.1%	
	Very good	1	20.0%	199	40.2%	
	Excellent	2	40.0%	89	18.0%	

Table 6 presents the factors influencing attitudes toward C-section among participants. A significant association was found between multiparous women and negative attitudes toward C-section, achieving statistical significance (*p*=.017). Participants who preferred vaginal delivery were significantly less inclined to view C-section favorably (*p*=.007). Moreover, participants self-assessing their knowledge as excellent exhibited a significantly higher tendency toward negative views on C-section compared to those rating their knowledge as poor, good, or very good (*p*=.003).

**Table 6:** Factors associated with attitudes of participants toward cesarean section *P-values based on the chi-squared test; statistical significance at *p*<0.05

n=500		Attitude toward cesarean section				
		Negative attitude		Positive attitude		
		N	%	N	%	*p*-value
Age (in years)	18-25	26	22.0%	114	29.8%	0.144
	26-30	10	8.5%	31	8.1%	
	31-35	12	10.2%	31	8.1%	
	36-40	19	16.1%	52	13.6%	
	41-45	28	23.7%	57	14.9%	
	>45	23	19.5%	97	25.4%	
Marital status	Single	22	18.6%	101	26.4%	0.218
	Married	91	77.1%	264	69.1%	
	Divorced/widowed	5	4.2%	17	4.5%	
Education	Below high school	2	1.7%	11	2.9%	0.315
	High school	16	13.6%	62	16.2%	
	Diploma degree	8	6.8%	43	11.3%	
	University degree	92	78.0%	266	69.6%	
Employment status	Unemployed	58	49.2%	195	51.0%	0.719
	Employed	60	50.8%	187	49.0%	
Family income	<5000	13	11.0%	30	7.9%	0.494
	5000-10000	42	35.6%	131	34.3%	
	>10000	63	53.4%	221	57.9%	
Parity	Nullipara	24	20.3%	120	31.4%	0.017^*^
	1	4	3.4%	25	6.5%	
	≥2	90	76.3%	237	62.0%	
Preferred mode of delivery	Vaginal delivery	101	85.6%	195	51.0%	<.001^*^
	Cesarean section	5	4.2%	95	24.9%	
	Neutral	12	10.2%	92	24.1%	
Participants' perceived knowledge rating	Poor	3	2.5%	40	10.5%	0.003^*^
	Good	39	33.1%	127	33.2%	
	Very good	44	37.3%	156	40.8%	
	Excellent	32	27.1%	59	15.4%	

## Discussion

The increasing prevalence of C-sections has become a significant concern in the global public health domain and among the medical community [14]. Childbirth is of great importance in women's lives, profoundly impacting their mental and social well-being, as well as that of their families [15]. Therefore, this study aimed to assess the knowledge and attitude toward modes of delivery and possible complications among women in the Al-Baha region, Saudi Arabia. By examining their perceptions and understanding, we sought to augment existing literature on this topic and offer valuable insights specific to the Al-Baha region. In this study, the majority of women preferred vaginal delivery (59%) over C-section (20%). These results align with several studies in various regions, including southern Ethiopia [16], eastern Ethiopia [17], California [18], Nepal [19], and the United Kingdom [20], all reporting a higher preference for vaginal delivery, with rates ranging from 69% to 93%. Specifically, these studies showed preferences of 87% (southern Ethiopia), 71.1% (eastern Ethiopia), 90.8% (California), 93% (Nepal), and 69% (United Kingdom), respectively. However, our findings contrast with a 2016 study from Iran, in which 62.9% of 200 pregnant women preferred C-section as their mode of delivery [21].

Furthermore, one factor influencing maternal preference for the mode of delivery was the history of pregnancy. In our study, a history of pregnancy was associated with a negative attitude toward C-section. This result is in line with a study conducted in eastern Ethiopia, where 146 out of 204 multigravida women preferred vaginal delivery [17]. Conversely, contrasting results were observed in southern Ethiopia, where mothers with multiple pregnancies had a higher preference for C-sections [16]. Additionally, studies in different countries have revealed diverse preferences for the mode of delivery based on parity. In California, about half (45%) of the nulliparous participants preferred vaginal delivery [18]. This suggests a tendency for a more vaginal delivery experience among first-time mothers in that context. In contrast, studies in the United Kingdom showed that 69% of primigravida preferred vaginal delivery despite the risk of obstetric anal sphincter injury (OASI). However, their preference shifted toward C-section as the severity of the tear increased, indicating a consideration of potential complications [20]. 

Regarding the knowledge rating, most participants in this study rated their knowledge as very good, accounting for 40.0%. Regionally, two studies in Egypt (Benha City and Cairo City) provided insightful data. The first study indicated that 46.3% of participants had a fair level of knowledge about the indications, advantages, and complications of both delivery modes. In contrast, the second study identified a significant information gap about various delivery methods [15][22]. Additionally, research conducted in Palestine highlighted that most participants were aware of the multiple complications associated with C-section delivery, with approximately 68% possessing an intermediate understanding of various delivery methods [23]. Similarly, a study in Iraq demonstrated that about 50% of pregnant women had good knowledge of delivery modes, with most acquiring their information from relatives [14]. In contrast, research in the United Arab Emirates revealed a notable lack of understanding regarding delivery modes, with around 78.4% of participants, particularly among the young and those with previous C-section deliveries, showing limited awareness [24]. Focusing on attitudes and preferences, 35% of women in Benha City, Egypt, exhibited a negative attitude toward C-section, a higher rate compared to 23.6% in this study [15]. Meanwhile, a survey in Cairo City found that, while 33.4% did not prefer C-section to avoid the lithotomy position, 72.5% expressed no regret after undergoing C-section [22]. In Palestine, a majority believed that the decision for C-section should align with the doctor's recommendation [23]. In Baghdad, the positive attitude toward C-section among pregnant women was 62%, closely aligning with the 76.4% observed in this study [14]. Finally, a study in the United Arab Emirates reported that approximately 86% of participants preferred vaginal delivery for their upcoming delivery [24]. 

At the national level, the knowledge outcomes of this study align with those of a study conducted in Jeddah, which found that a majority of pregnant women, 78.2%, possessed adequate knowledge and only 7.3% exhibited insufficient knowledge about complications [25]. Conversely, our results diverge from a more recent Jeddah study, indicating that most participants, 45.4%, had poor knowledge regarding complications, with only 12.6% demonstrating good knowledge. However, in our study, more than half of the participants (55.6%) exhibited sufficient knowledge about postpartum complications. Additionally, the latter study observed that 44.1% of women with university education had poor knowledge of C-section complications. In contrast, our study indicated that women holding a university degree demonstrated a higher level of understanding regarding postpartum complications [26]. Furthermore, research conducted in the Makkah region yielded results consistent with ours, revealing that around half of the primigravida mothers had a fair knowledge of postpartum complications (48%) [27]. Regarding attitudes, the current study showed that 99% of participants had a positive attitude toward vaginal delivery and approximately 76% had a positive attitude toward C-section. In contrast, a study in Jeddah found that 66.5% of pregnant women had a negative attitude toward elective C-section (ECS) [25]. Similarly, another Jeddah study concluded that the majority of participants (52%) held negative views toward C-section and preferred vaginal delivery [26]. Correspondingly, a study in Makkah found that over half of the primigravida mothers had a positive attitude toward vaginal delivery (68%) [27].

Limitations

Our study, employing a cross-sectional design, cannot establish a cause-and-effect relationship nor identify the exact reasons for either favorable or negative attitudes toward delivery methods. Moreover, the findings of our study are not universally applicable to all Saudi women. Additionally, it's important to note that there is a tendency for patients who delivered 3-5 years ago to forget their experiences, which may affect their ability to recall and accurately report their attitudes toward delivery methods.

## Conclusions

This study examined the awareness, knowledge, and attitudes of women in Al-Baha, Saudi Arabia, toward various modes of delivery and their understanding of potential postpartum complications. It was found that over half of the participants had adequate knowledge about postpartum complications and the majority held a positive view of vaginal delivery. Significantly higher knowledge levels regarding postpartum complications were noted among participants with a university degree compared to those with only a diploma or lesser qualification. Additionally, those who preferred C-section exhibited a markedly lower understanding of postpartum complications than those favoring vaginal delivery. However, participants with a history of pregnancy who preferred vaginal delivery and self-rated their knowledge as excellent were more inclined to view C-section negatively. We advocate for the implementation of specific guidelines in healthcare facilities concerning the indications for C-section. We also endorse the provision of comprehensive information by medical professionals about various delivery methods and their complications, indications, and benefits to pregnant women. Furthermore, we suggest that future research should explore the levels of knowledge and attitudes regarding modes of delivery and associated complications on a national scale. 

## Appendices

Questionnaire

Section 1

Age group:

18-25 years old

30-26 years old

35-31 years old

40-36 years old

45-41 years old

Over 45 years old

Marital status:

Single

Married

Divorced

Widowed

Education:

I never studied

Elementary/middle school

High school

Diploma

Higher-level education (bachelor's, master's, doctorate)

Occupation:

Employee

Not employed

Have you ever been pregnant?

I have never been pregnant

Once

Multiple times

What is your preferred birth method?

Vaginal delivery

Birth via cesarean section

I don't know

Monthly family income:

Less than 5000 Saudi riyals

From 5,000 to 10,000 Saudi riyals

More than 10,000 Saudi riyals

How do you evaluate your knowledge about birth methods and possible complications?

Poor

Good

Very good

Excellent

Section 2 (Dedicated to Assessing Knowledge Toward Vaginal Birth and Determining the Attitude Toward It)

Vaginal delivery is a natural and acceptable method

Strongly agree

Agree

Neutral

Disagree

Strongly disagree

After vaginal delivery, the mother's happiness increases after seeing her baby

Strongly agree

Agree

Neutral

Disagree

Strongly disagree

The recovery period is quick after vaginal delivery

Strongly agree

Agree

Neutral

Disagree

Strongly disagree

After vaginal delivery, the emotional relationship between the mother and the infant is better

Strongly agree

Agree

Neutral

Disagree

Strongly disagree

Vaginal delivery spares the mother the high risks of anesthesia and the risks of undergoing surgery

Strongly agree

Agree

Neutral

Disagree

Strongly disagree

Vaginal delivery is better in the long term

Strongly agree

Agree

Neutral

Disagree

Strongly disagree

I prefer vaginal delivery to avoid scarring on the abdomen

Strongly agree

Agree

Neutral

Disagree

Strongly disagree

Complications of vaginal delivery are less compared to cesarean section

Strongly agree

Agree

Neutral

Disagree

Strongly disagree

Section 3 (Dedicated to Assessing Knowledge Regarding Cesarean Section)

Birth by cesarean section is better than vaginal birth

Strongly agree

Agree

Neutral

Disagree

Strongly disagree

I prefer a cesarean section to avoid the stress associated with a vaginal birth

Strongly agree

Agree

Neutral

Disagree

Strongly disagree

I prefer a cesarean section because I don't want to go through labor pain

Strongly agree

Agree

Neutral

Disagree

Strongly disagree

Giving birth to a baby by cesarean section is considered healthier

Strongly agree

Agree

Neutral

Disagree

Strongly disagree

A cesarean section is better because a tubal ligation can be performed at the same time

Strongly agree

Agree

Neutral

Disagree

Strongly disagree

Cesarean section is better because it prevents prolapse of the bladder and uterus

Strongly agree

Agree

Neutral

Disagree

Strongly disagree

Cesarean section is considered better because it prevents deformation and tearing of the reproductive system

Strongly agree

Agree

Neutral

Disagree

Strongly disagree

I prefer a cesarean section despite its possible complications

Strongly agree

Agree

Neutral

Disagree

Strongly disagree

A cesarean section must be performed according to the mother's wishes

Strongly agree

Agree

Neutral

Disagree

Strongly disagree

Cesarean section should be performed when vaginal delivery is too risky

Strongly agree

Agree

Neutral

Disagree

Strongly disagree

Section 4 (Dedicated to Assessing Knowledge of Postpartum Complications)

The range of postpartum complications ranges from relatively minor and temporary to life-threatening conditions which can be divided into seven categories:

Childbirth can lead to infections such as endometriosis

Strongly agree

Agree

Neutral

Disagree

Strongly disagree

Childbirth can lead to deep vein thrombosis

Strongly agree

Agree

Neutral

Disagree

Strongly disagree

Childbirth can lead to bleeding

Strongly agree

Agree

Neutral

Disagree

Strongly disagree

A cesarean section can lead to a hematoma in the bladder

Strongly agree

Agree

Neutral

Disagree

Strongly disagree

A cesarean section can lead to wound infection

Strongly agree

Agree

Neutral

Disagree

Strongly disagree

Childbirth can lead to complications resulting from a medical error, such as uterine perforation

Strongly agree

Agree

Neutral

Disagree

Strongly disagree

Childbirth can lead to various complications, such as acute cholecystitis

Strongly agree

Agree

Neutral

Disagree

Strongly disagree

## References

[1] Torloni MR, Betrán AP, Montilla P (2013). Do Italian women prefer cesarean section? Results from a survey on mode of delivery preferences. BMC Pregnancy Childbirth.

[2] Baskett TF, Calder AA, Arulkumaran S (2014). Munro Kerr's operative obstetrics. Edinburgh: Saunders Elsevier.

[3] Guittier MJ, Cedraschi C, Jamei N, Boulvain M, Guillemin F (2014). Impact of mode of delivery on the birth experience in first-time mothers: a qualitative study. BMC Pregnancy Childbirth.

[4] Betran AP, Ye J, Moller AB, Souza JP, Zhang J (2021). Trends and projections of caesarean section rates: global and regional estimates. BMJ Glob Health.

[5] (2018). Dewhurst's textbook of obstetrics & gynaecology. https://search.worldcat.org/title/1029070067.

[6] Mascarello KC, Matijasevich A, Santos ID, Silveira MF (2018). Early and late puerperal complications associated with the mode of delivery in a cohort in Brazil. Rev Bras Epidemiol.

[7] Sharma S, Dhakal I (2018). Cesarean vs vaginal delivery : an institutional experience. JNMA J Nepal Med Assoc.

[8] Weckesser A, Farmer N, Dam R, Wilson A, Morton VH, Morris RK (2019). Women's perspectives on caesarean section recovery, infection and the PREPS trial: a qualitative pilot study. BMC Pregnancy Childbirth.

[9] Majlesi M, Montazeri A, Rakhshani F, Nouri-Khashe-Heiran E, Akbari N (2020). 'No to unnecessary caesarean sections': evaluation of a mass-media campaign on women's knowledge, attitude and intention for mode of delivery. PLoS One.

[10] Betran AP, Torloni MR, Zhang J (2015). What is the optimal rate of caesarean section at population level? A systematic review of ecologic studies. Reprod Health.

[11] Molina G, Weiser TG, Lipsitz SR (2015). Relationship between cesarean delivery rate and maternal and neonatal mortality. JAMA.

[12] Declercq E, Young R, Cabral H, Ecker J (2011). Is a rising cesarean delivery rate inevitable? Trends in industrialized countries, 1987 to 2007. Birth.

[13] (2023). Sample size calculator. http://www.raosoft.com/samplesize.html.

[14] Nasir NA, Amir H (2017). Knowledge and attitude of pregnant women towards modes of delivery in an antenatal care clinic in Baghdad. J Fac Med Baghdad.

[15] Alkalash S, El Kelany O, Zayed M (2021). Cesarean sections rate and maternal knowledge and attitude towards the mode of delivery in Egypt. Menoufia Medical Journal.

[16] Tenaw Z, Kassa ZY, Kassahun G, Ayenew A (2019). Maternal preference, mode of delivery and associated factors among women who gave birth at public and private hospitals in Hawassa City, Southern Ethiopia. Ann Glob Health.

[17] Welay FT, Gebresilassie B, Asefa GG, Mengesha MB (2021). Delivery mode preference and associated factors among pregnant mothers in Harar Regional State, Eastern Ethiopia: a cross-sectional study. Biomed Res Int.

[18] Yee LM, Kaimal AJ, Houston KA (2015). Mode of delivery preferences in a diverse population of pregnant women. Am J Obstet Gynecol.

[19] Joshi A, Thapa M, Panta OB (2018). Maternal attitude and knowledge towards modes of delivery. J Nepal Health Res Counc.

[20] Long E, Jha S (2018). Factors that influence patient preference for mode of delivery following an obstetric anal sphincter injury. Eur J Obstet Gynecol Reprod Biol.

[21] Zamani-Alavijeh F, Araban M, Hassanzadeh A, Makhouli K (2018). Contributing factors of pregnant women's beliefs towards mode of delivery: a cross-sectional study from Iran. Matern Health Neonatol Perinatol.

[22] Alaa-El-Din Wali A, Taher A, Abd-El-Fatah SM (2020). Awareness, knowledge, and attitude of Egyptian women toward cesarean delivery: a cross-sectional survey. J South Asian Feder Obst Gynae.

[23] Samara B, Sabella AR (2021). The knowledge and attitudes of Palestinian women towards different childbirth delivery options. Clin Exp Obstet Gynecol.

[24] Al-Rifai RH, Elbarazi I, Ali N, Loney T, Oulhaj A, Ahmed LA (2020). Knowledge and preference towards mode of delivery among pregnant women in the United Arab Emirates: the Mutaba'ah study. Int J Environ Res Public Health.

[25] Al Sulamy AA, Yousuf SA, Thabet HA (2019). Knowledge and attitude of pregnant women toward elective cesarean section in Saudi Arabia. Open J Nurs.

[26] Yaqoub RM, Khouj MA, Alsaif AA, Eissa GA, Alhemdi JA, Albasri S (2022). Awareness and knowledge of caesarean section complications among women in Jeddah, Saudi Arabia. Cureus.

[27] Faden AA, Alahmadi AS, Bogis MM (2022). Primigravida mothers' knowledge and attitude towards vaginal delivery and caesarean section. Ann Rom Soc Cell Biol.

